# Dual psychometric evaluation of measures assessing attitudes toward prenatal alcohol exposure and fetal alcohol spectrum disorder

**DOI:** 10.1111/acer.70239

**Published:** 2026-02-09

**Authors:** Ruth H. Brown, Stewart McDougall, Suzanne O'Rourke

**Affiliations:** ^1^ Health in Social Sciences Department University of Edinburgh Edinburgh UK

**Keywords:** attitudes, fetal alcohol spectrum disorder, prenatal alcohol use, psychometric measure

## Abstract

**Background:**

Uninformed attitudes toward prenatal alcohol exposure (PAE) and fetal alcohol spectrum disorder (FASD) remain barriers in the assessment and diagnosis of affected individuals. While there is a growing need to evaluate the attitudes of professionals who work closely with pregnant individuals and those living with FASD, it is currently unknown whether existing psychometric tools, quantifying such attitudes, are reliable. The psychometric properties of two measures were therefore investigated: the “Alcohol and Pregnancy Measure” (capturing attitudes toward PAE) and the “Knowledge and Attitudes Regarding FASD Measure” (capturing attitudes toward FASD).

**Methods:**

A total of 1797 healthcare workers, recruited largely from the National Health Service in Scotland, completed both psychometric measures as part of a nationwide survey of attitudes. Participants also completed novel measurements of their knowledge of FASD and their attitudes toward the health advice for pregnant women regarding alcohol use during pregnancy.

**Results:**

Support for the psychometric validity of the two measures was partially observed. Both measures were found to have multifactor structures, instead of the hypothesised one‐factor solutions. Such multifactor structures demonstrated goodness‐of‐fit in confirmatory factor analyses. Moreover, both measures showed convergent validity with both knowledge of FASD and attitudes toward current health advice regarding PAE. Both the measures' sensitivity to ceiling effects and poor subfactor internal consistencies, however, remain a concern.

**Conclusion:**

Results indicate that both measures, when used at the total‐scale level, can acceptably assess attitudes toward PAE and FASD. Revisions are required at the item‐level to further improve subfactor internal consistency and minimize ceiling effect likelihood.

## Graphical abstract

Uninformed attitudes toward prenatal alcohol exposure and fetal alcohol spectrum disorder (FASD) hinder assessment and diagnosis. The reliability of two attitude measures—the Alcohol and Pregnancy Measure and the Knowledge and Attitudes Regarding FASD Measure—was assessed using data from 1797 healthcare workers. Both measures showed multifactor structures with good model fit and convergent validity with FASD knowledge and attitudes toward prenatal alcohol use guidance. While total scales were reliable, several subfactors demonstrated poor internal consistency.

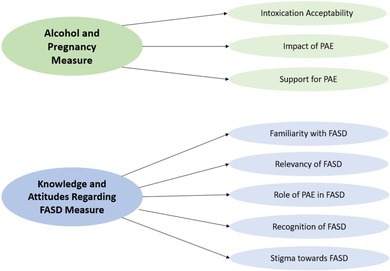

Uninformed or potentially stigmatizing attitudes toward prenatal alcohol exposure (PAE) and fetal alcohol spectrum disorder (FASD)—held by criminal justice, health, and social care professionals—have been routinely noted as a significant barrier in the assessment and diagnosis of FASD (Corrigan et al., 2019; Dunbar Winsor, 2021; McCormack et al., 2022), and the provision of support to those who consume alcohol during pregnancy (Aspler et al., 2021). Indeed, caregivers of children with FASD have highlighted the obligation to advocate for their child's needs when working alongside professionals, noting negative experiences due to professionals’ reluctance to both diagnose and ask about PAE history, likely due to fear of “labelling” the child and “blaming” the mother (Bell et al., 2016; Dunbar Winsor, 2021; Wilson et al., 2024). Professionals themselves have reported being underprepared and undersupported to provide formal help to those affected by PAE/FASD, with both a fear of stigmatizing individuals with FASD (Wilson et al., 2024) and a general lack of confidence in evaluating alcohol use in pregnant individuals (Smith et al., 2021) highlighted in previous literature.

Provision of bespoke training, providing knowledge and targeting potentially underlying misinformed beliefs about PAE and FASD, may address and ameliorate such barriers. Indeed, international literature from North America, Australia, and New Zealand has reported the success of such training opportunities to change the attitudes and knowledge of professionals toward PAE/FASD (McCormack et al., 2022). For example, FASD‐specific training provided to Australian healthcare workers was noted to have positive effects on their practice, with higher confidence in both asking about pregnancy and in contributing to the diagnosis of FASD, a key outcome reported by the trainees (Reid et al., 2020). Moreover, provision of FASD‐specific training to those working within the criminal justice system was found to significantly improve justice professionals' knowledge of the condition, with a large proportion of the trainees indicating that they would be more empathetic and patient with youth offenders post‐training, in turn improving the quality of support (Passmore et al., 2020).

However, the large majority of previous studies investigating the effectiveness of such training packages did not administer previously validated psychometric measures to quantify change in the professionals' attitudes, instead relying on qualitative testimonies (e.g., Reid et al., 2020) or novel, unvalidated questionnaires to evaluate training success (e.g., McCormack et al., 2022). While broadly useful to gauge the training's impact, the lack of standardized measures of attitudes is a critical limitation of past literature. Specifically, without routinely applied and psychometrically validated measures, comparability across studies is limited, thus greatly reducing the opportunity to generate robust, comparative evidence of training effectiveness. Use of standardized measurements instead would offer the ability to meaningfully monitor attitudinal change over time, across both professional sectors and different training packages.

Two previously developed measures may be suitable candidates to bridge this gap. First, based on a survey developed by Health Canada (Environics Research Group Limited, 2000), the “Alcohol and Pregnancy Measure,” created by Peadon et al. (2011) was developed to capture attitudes toward alcohol use during pregnancy. Participants are asked to read statements and rate to what extent they align with their beliefs (e.g., “The more alcohol a pregnant women drinks, the more likely that the unborn child will be affected”). The measure has been previously used to evaluate change in attitudes toward PAE after engaging with an educational intervention aimed at pregnant individuals (Keating et al., 2025). Second, the “Knowledge and Attitudes Regarding FASD Measure,” developed by Passmore et al. (2018), was originally developed to assess attitudes toward FASD in criminal justice professionals based in Australia. The scale was cocreated with custodial and clinical staff, as well as FASD researchers, including both Aboriginal and non‐Aboriginal professionals (Passmore et al., 2018). Similar to Peadon et al.'s measure, participants are asked to rate statements pertaining to FASD on what extent they agree with them (e.g., “FASD occurs primarily in financially disadvantaged families”). Until now, the “Knowledge and Attitudes Regarding FASD Measure” has not been utilized outside of its original development.

To date, however, neither measure has undergone psychometric validation either during their development or subsequently, and it therefore remains unclear whether either tool can be used to reliably assess attitudes toward PAE and FASD. Moreover, outside of Keating et al. (2025) use of the full “Knowledge and Attitudes Regarding FASD Measure,” both measures have only been used at the item level; thus, it is unclear whether the items can be summed together to produce a single, interpretable score, nor is it known whether meaningful subfactors can be extracted. Using a large sample of health and social care professionals, recruited across all NHS Scotland health boards, this study therefore aimed to:

- assess the underlying factor structure of both measures to determine whether a single‐factor structure provides the best fit (“baseline” factor structure), or whether alternative structures with multiple subfactors emerge (“optimized” factor structure);
- assess the goodness‐of‐fit of both the single‐factor “baseline” and multifactor “optimized” structures of both measures; and
- to evaluate the strength of convergent validity with knowledge of FASD and attitudes toward the current alcohol abstinence advice for pregnant women.

## METHODS

### Participants

Participants were pooled from two separate data collection periods. The first sample came from an evaluation of a novel training webinar, *the Fundamentals of FASD*, on improving knowledge and attitudes toward PAE and FASD in professionals from Scotland's health and social care sectors (*n* = 1005; McDougall et al., n.d.). The second sample came from a national evaluation of knowledge and attitudes within Scotland's health and social care professionals (*n* = 1018; McDougall et al., 2025). For both study populations, inclusion criteria were: (i) aged 16 and above, (ii) residing within Scotland, (iii) able to read and speak English, and (iv) currently working within NHS Scotland or Public Sector Social Care Services. Participants in the second sample were asked about previous training experiences, including whether they had attended the training evaluated in the first sample. Participants who indicated they had attended the training were removed from the sample to minimize potential overlap.

### Procedure

For the first sample (McDougall et al., n.d.), participants who expressed an interest in attending *the Fundamentals of FASD* webinar were sent an anonymous online questionnaire prior to the training event to quantify the trainees' baseline attitudes and knowledge toward PAE/FASD. The webinar was advertised on mailing lists of the NHS and social care in Scotland, as well as via social media. Participants who were interested in attending the webinar were asked to initially register to attend via the Eventbrite page, and were then sent instructions on how to access the webinar as well as the pretraining evaluation questionnaire. For the second sample (McDougall et al., 2025), directors of communication for each of the NHS Scotland health boards disseminated a recruitment flyer, inviting participants to take part in a similar online questionnaire used in the first wave of data collection, via email to all staff across all NHS Scotland health boards and Social Care Partnerships. As it was not feasible to establish the number of, a response rate was unable to be computed. By way of increasing uptake, participants in the second sample were invited to enter into a raffle to win one of four gift cards.

Across both studies, questionnaires were hosted on the GDPR‐compliant survey software, *Qualtrics*. All participants provided informed consent prior to engaging with the questionnaire and were debriefed of the study's aims upon completion. Both studies received ethical approval from the University of Edinburgh's Health in Social Sciences Research (Ethics Committee; McDougall et al., n.d., CLPS208; McDougall et al., 2025, CLPS261).

### Materials

Data on participant demographics; attitudes toward (i) PAE, (ii) FASD, and (iii) the current health advice regarding alcohol abstinence during pregnancy; and knowledge of FASD were extracted and merged from the datasets of the previous studies (McDougall et al., n.d., 2025). The order of the psychometric measures within the questionnaires, as presented to the participants, was consistent across the two samples. A summary of the measures is provided below.

#### Alcohol and Pregnancy Measure (Peadon et al., 2011)

To evaluate attitudes toward prenatal alcohol exposure, the 12‐item “Alcohol and Pregnancy Measure,” developed by Peadon et al. (2011) was used. Items are rated from 1 = “Strongly Disagree” to 5 = “Strongly Agree,” with higher scores indicative of more informed attitudes toward PAE. Two items within the scale, “it is ok for pregnant women to drink three or four units of alcohol in one day,” and “it is ok for pregnant women to become intoxicated” are reverse‐coded to allow all items to align with the same attitudinal direction.

#### Knowledge and Attitudes Regarding FASD Measure (Passmore et al., 2018)

To evaluate attitudes toward FASD, the original 17‐item “Knowledge and Attitudes Regarding FASD Measure” (Passmore et al., 2018), developed to capture the knowledge and attitudes toward FASD in Western Australian youth custodian workers was used. Statements are rated on a 5‐point Likert scale, from 1 = “Strongly Disagree” to 5 = “Strongly Agree.” In both studies, the original items, “FASD occurs primarily in Aboriginal families,” and “FASD is relevant to the justice system” were removed to make the measure more cross‐culturally compatible. Moreover, two additional items, “FASD is relevant to my work at BHDC” and “I am familiar with how FASD might affect young people and adults involved in the criminal justice system” were amended to become, “FASD is relevant to my work,” and “I am familiar with the difficulties people with FASD can experience,” respectively. Finally, one additional item was added by the research team; “The emphasis on FASD is stigmatising to women.” Nine items within the scale (e.g., “People can grow out of FASD”) are reverse‐coded to allow all items to align with the same attitudinal direction. Total scores ranged from 16 to 80, with higher scores indicative of more informed attitudes toward FASD.

#### Additional measures

##### Attitudes toward health advice

A three‐item scale measuring participants' attitudes toward the current Chief Medical Officer's health advice regarding alcohol abstinence during pregnancy (Chief Medical Officer, 2016) was developed by the research team (Table S1). Items (e.g., “there is no safe level of alcohol during pregnancy”) were rated from 1 = “Strongly Disagree” to 5 = “Strongly Agree”, with scores ranging from 5 to 15. Higher scores were indicative of stronger endorsement of the alcohol abstinence message.

##### Knowledge of FASD

To assess the participants' knowledge of key facts about FASD, a 12‐item measure was developed by the research team (Table S2). The measure consisted of a mixture of true/false (e.g., “True or false, FASD is found across the socio‐economic status spectrum?”) and multiple choice (e.g., “What is the estimated prevalence of FASD in Scotland?”) questions. Scores ranged from 0 to 12, with higher scores indicative of better knowledge. Given the variance in how items were scored (i.e., scored dichotomously for the true/false questions and fractionally scored for the multiple‐choice questions), internal consistency was not generated for this measure.

### Statistical analysis

After merging the data, data cleaning, and investigating the total sample participant demographics, the total sample (*n* = 1797) was randomized using a random number generator and dichotomised at the 898th participant to give two similarly weighted groups, with one group's data used to conduct exploratory factor analyses (“EFA group”; *n* = 899), and the latter group's data used to conduct confirmatory factor analyses (“CFA group”; *n* = 898). This was conducted to avoid running EFA and CFA on the same sample (Finch, 2020). Moreover, several CFAs can be conducted on the same data (Finch, 2020); thus, the goodness‐of‐fit for both baseline one‐factor structures and optimized factor structures was tested.

Second, EFAs were conducted on the EFA group (*n* = 899) in order to explore the underlying factor structures for the PAE and FASD attitude measures. Both a Kaiser–Meyer–Olkin (KMO) test of sampling adequacy value above 0.600, and a significant Bartlett's test of sphericity were used to determine whether the data were suitable for factor analysis (Shrestha, 2021). Given it was predicted that the subfactors would correlate, an oblimin rotation with Kaiser normalization was used. Moreover, maximum likelihood was used instead of other extraction methodologies (e.g., principal components analysis) as per the recommendations of Costello and Osborne (2005). Extracted factors with eigenvalues >1.00 were deemed meaningful (Kaiser, 1960). Items with factor loadings <0.300 were deemed to have weak conceptual alignment, limiting the parsimony of the measures' items; thus, such items were excluded from the item pool (Costello & Osborne, 2005). Given that ceiling effects were observed across the two measures, both at the total scale (see Table 1) and item‐level (see Supplementary Tables 3 and 4), the weighted least squares mean and variance adjusted (WLSMV) estimator was utilized. Such estimation method is recommended over others (e.g., maximum likelihood), especially when the data are skewed and/or ordinal (Li, 2016).

Third, to establish the model‐fit of the baseline and optimized factor structures identified in the EFA, a series of CFA models were conducted on the CFA group's data (*n* = 898). Model fit was considered adequate if the following absolute fit indices were met: (i) comparative fit index (CFI) and Tucker–Lewis index (TLI) > 0.900, (ii) root mean square error of approximation (RMSEA) < 0.060, and (iii) standardized root mean square residual (SRMR) < 0.080 (Sun, 2005). As the sample sizes used in this study were large, it was predicted that model chi‐squared values would emerge as significant, given the test's sensitivity toward sample size (Kyriazos, 2018). Improved model fit was identified by changes in BIC (ΔBIC) over 10 (Bollen et al., 2014; Raftery, 1995). Similar to the EFAs, WLSMV estimation was utilized in the CFA models. Exploratory post hoc modification indices were lastly run to identify whether residual covariances were present and thus if freeing constrained parameters could improve model fit (i.e., to allow additional covariances between the residuals to be estimated, rather than fixed to zero). While a MI cut‐off value of 3.84 is often cited, such cutoff has been criticized. For example, past authors have highlighted that overuse of MI's (i.e., freeing all parameters that are above the 3.84 threshold) often leads to poor model identification and overfitting; thus, more conservative cutoffs and more theory‐driven approaches are recommended (Whittaker, 2012). Here, a cutoff of >20 was applied to identify candidate parameters. This was done to both ensure only meaningful and theoretically justifiable modifications were added to the exploratory models (e.g., between two semantically identical items), and to avoid artificially inflating model fit (Byrne, 2010).

Lastly, interfactor correlations and internal consistencies across the two measures were assessed. Correlations were classified as small (<0.300), medium (<0.500) and large (>0.500), using recommended cutoff values to determine correlational effect size (Cohen, 1988). Moreover, internal consistency was considered acceptable with Cronbach alpha values >0.700 (Lance et al., 2006). Lastly, convergent validity of the two measures was evaluated by correlating scores with two novel measures developed by the research team; (i) a three‐item measure assessing attitudes capturing attitudes toward the current health advice regarding alcohol use during pregnancy; and (ii) a 12‐item measure created to assess participant knowledge of FASD. Analyses were conducted on both SPSS version 27 and the lavaan package for R (Rosseel, 2012).

## RESULTS

### Initial data cleaning

A total of 2023 participants consented to participate. On inspection of the dataset, 50 participants provided consent but did not complete any sections of the questionnaire and were thus removed from the dataset. A further 143 participants were removed as they did not complete full sections of the questionnaire beyond the participant demographics section (72 removed for not completing any of the measures; 28 removed for completing only one measure, and 43 removed for only completing two measures), giving a sample size of 1830. A total of 33 participants were found to have completed the measures twice due to participating in both data collection waves, and were thus excluded, giving a final sample of 1797. Lastly, as 9.62% of the data were found to be missing, a Little's MCAR test was conducted, indicating that the data were not missing completely at random (*χ* ^2^ (1360) = 1845.36, *p* < 0.001). Missing data were therefore handled by the fully conditional specification method; generating a single dataset for analysis. Lastly, normality of the total score data was assessed via Kolmogorov–Smirnoff and Shapiro–Wilks tests. Both the Alcohol and Pregnancy Measure and Knowledge and Attitudes Regarding FASD scales were found to have significant Kolmogorov–Smirnoff (K‐S [1797] = 0.157, *p* < 0.001 and K‐S [1797] = 0.048, *p* < 0.001; respectively) and Shapiro–Wilk (S‐W [1797] = 0.717, *p* < 0.001 and S‐W [1797] = 0.986, *p* < 0.001; respectively), thus indicating non‐normality. Spearman's rho (*ρ*) was therefore utilized for the correlational analyses.

### Sample demographics

A full overview of the psychometric measure metrics and the total sample's demographics can be seen in Tables 1 and 2. In short, most of the measures demonstrated good internal consistency (e.g., *Alcohol and Pregnancy Measure*; *α* = 0.823); whereas the Knowledge of FASD scores were just below acceptable levels (i.e., *α* = 0.672). Moreover, ceiling effects were observed across all measures, whereby the mean scores of all the measures were close to the highest score possible.

**TABLE 1:** Psychometric measure metrics.

Psychometric measure	*α*	Mean	SD	Range	Skewness	Kurtosis
Alcohol and Pregnancy Measure	0.823	51.37	6.71	12–60	−3.13	13.54
Knowledge and Attitudes Toward FASD Measure	0.788	58.29	7.41	17–77	−0.404	1.17
Attitudes toward health advice	0.835	13.42	2.47	3–16	−2.65	7.65
Knowledge of FASD	0.672	8.50	1.74	0–12	−1.13	2.90

**TABLE 2:** Overview of sample demographics.

Demographic variable	Total sample (*n* = 1797)	EFA sample (*n* = 899)	CFA sample (*n* = 898)	Group difference
**NHS Health Board**				
Ayrshire and Arran	75	33	42	*χ* ^2^ (16) = 15.26, *p* = 0.506
Borders	98	52	46	
Dumfries and Galloway	23	12	11	
Fife	79	39	40	
Forth Valley	113	59	54	
Grampian	198	103	95	
Greater Glasgow and Clyde	264	120	144	
Highland	138	73	65	
Lanarkshire	184	96	88	
Lothian	215	111	104	
Orkney	19	9	10	
Shetland	26	9	17	
Tayside	119	66	53	
Western Isles	81	47	34	
Other NHS Scotland	65	27	38	
Outside of NHS Scotland	58	24	34	
Missing	42	19	23	
**Professional background**				
Psychology	151	78	73	*χ* ^2^ (17) = 15.88, *p* = 0.530
Social Work	196	104	92	
Nursing	540	257	283	
General Practitioners	53	30	23	
Speech and Language Therapist	216	110	106	
Occupational Therapist	63	28	35	
Midwifery	11	4	7	
Pediatrics	6	3	3	
Psychiatry	22	14	8	
Educational Psychology	16	6	10	
Administrative Roles	72	34	38	
Paramedics	43	22	21	
Public Health	8	5	3	
Pharmacy	15	4	11	
Other	366	190	176	
Missing	19	10	9	
**Educational attainment**				
National 5/Standard Grade equivalent	54	24	30	*χ* ^2^ (6) = 6.74, *p* = 0.346
Highers/A‐levels	81	37	44	
Trade/technical/vocational training	77	47	30	
Bachelor's Degree	666	337	329	
Postgraduate Degree (e.g., Master's)	646	319	327	
Doctorate	103	56	47	
Other	165	77	88	
Missing	5	2	3	

Regarding the sample, the vast majority of the sample was recruited from NHS Scotland (*n* = 1697, 94.44%), with a large proportion of participants coming from nursing/midwifery (*n* = 551, 30.66%), allied health (*n* = 279, 15.52%), social work (*n* = 196, 10.91%), psychology/psychiatry (*n* = 189, 10.52%), and medical backgrounds (*n* = 59, 3.28%), with 366 participants (20.37%) coming from other health and social care sectors (e.g., research, health visitors, and rehabilitation support). Moreover, most participants had received some form of university education (*n* = 1415, 78.74%). The EFA (*n* = 899) and CFA (*n* = 898) groups were not found to differ significantly across the demographic variables.

### Step 1: Exploratory factor analyses

Using data from the EFA sample (*n* = 899), it was first of interest to identify the underlying factor structures of the PAE and FASD measures, as well as identify item(s) that were eligible for exclusion.

#### Underlying factor structure of the alcohol and pregnancy measure

**FIGURE 1:**
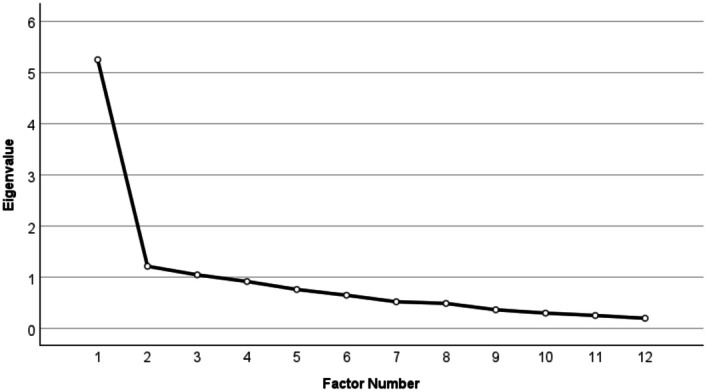
Scree plot of Alcohol and Pregnancy Measure.

The KMO test of sampling adequacy was 0.893 suggesting the data had high factorability; and the Bartlett's test of sphericity was significant (*χ* ^2^ [66] = 4795.80, *p* < 0.001), indicating sufficient strength of correlations between the items inserted into the model. Three subfactors were identified in the EFA model (Figure 1 and Table 3), with the total model explaining 62.61% of the variance, with “F1: Intoxication Acceptability” explaining 43.71%, “F2: Impact of PAE” explaining 10.15%, and “F3: Support for PAE” explaining 8.75% of the variance. The items, “Pregnant women should drink less than seven units each week” and “Women are aware of the effects that drinking alcohol during pregnancy can have on the unborn child” were found to have no loadings and were thus removed from further analyses (Table 3).

**TABLE 3:** Exploratory factor analysis on the Alcohol and Pregnancy Measure, with oblimin rotation and maximum likelihood extraction.

Item	*α* if item deleted	F1: Intoxication acceptability	F2: Impact of PAE	F3: Support for PAE
It is ok for pregnant women to drink three or four units of alcohol in 1 day^a^	0.784	0.870	*0.199*	*0.202*
It is ok for pregnant women to become intoxicated^a^	0.787	0.520	*0.205*	*0.316*
Drinking alcohol during pregnancy can affect the unborn child	0.774	*0.027*	1.04	*0.165*
Drinking alcohol can lead to life‐long disabilities in a child	0.776	*0.066*	0.822	*0.016*
The more alcohol a pregnant women drinks, the more likely that the unborn child will be affected	0.787	*0.000*	0.485	*0.133*
Information should be readily available about the effect that drinking alcohol may have on the unborn child	0.777	*0.053*	0.421	*0.214*
Pregnant women should not drink alcohol	0.785	*0.130*	0.415	*0.193*
Health professionals should advise women who are pregnant or who are thinking of becoming pregnant to give up drinking alcohol	0.782	*0.004*	*0.131*	0.722
Health professionals should ask pregnant women about how often they drink alcohol	0.779	*0.073*	*0.235*	0.495
Members of the general public are concerned about women drinking alcohol during pregnancy	0.805	*0.005*	*0.031*	0.401
*Pregnant women should drink less than seven units of alcohol each week*	0.864	*0.081*	*0.032*	*0.043*
*Women are aware of the effects that drinking can have on the unborn child*	0.826	*0.020*	*0.089*	*0.019*

*Note*: Items in italics had factor loadings <0.300.

^a^ Reverse‐scored items.

#### Underlying factor structure of the knowledge and attitudes regarding FASD measure

**FIGURE 2:**
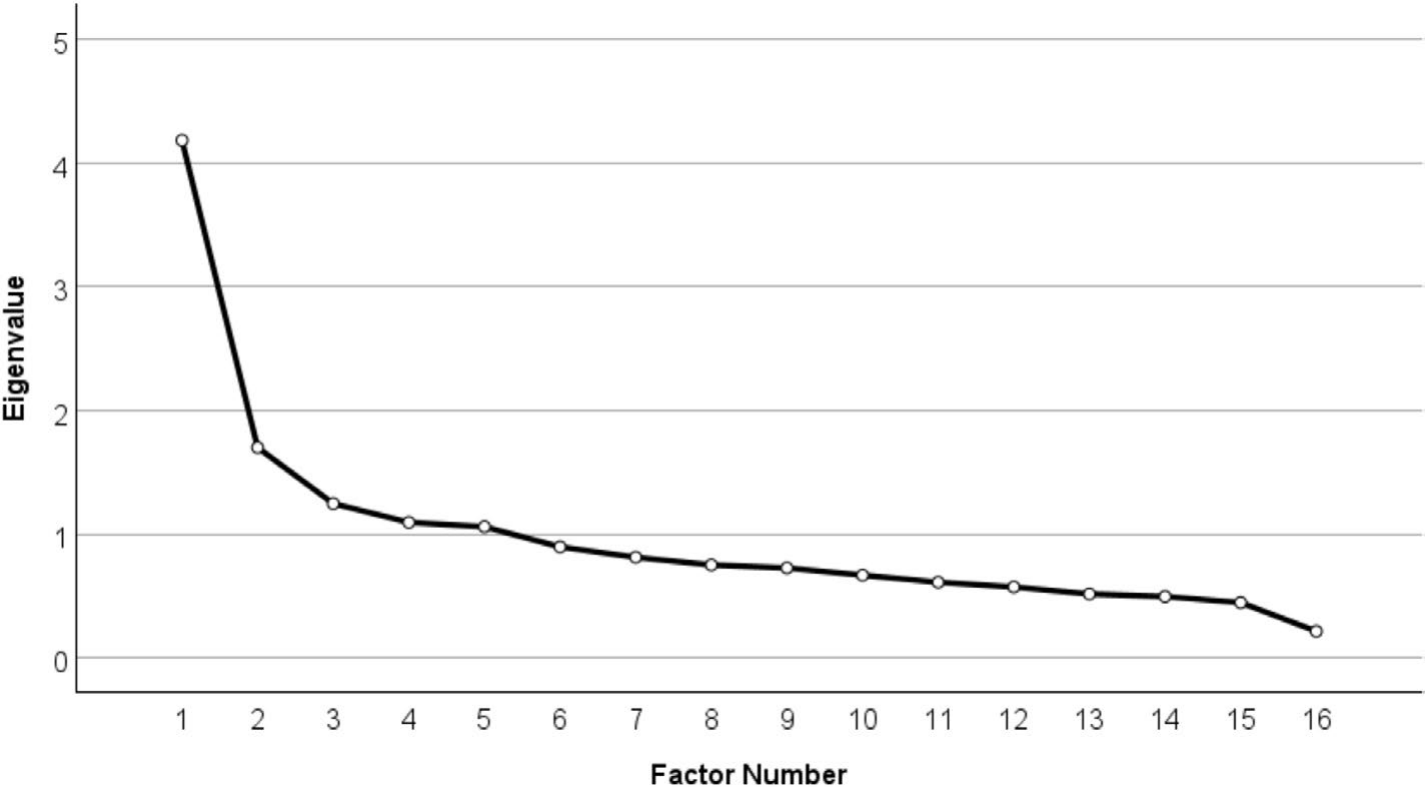
Scree plot of Knowledge and Attitudes Regarding FASD Measure.

The KMO test of sampling adequacy was 0.829, suggesting the data had high factorability; and the Bartlett's test of sphericity was significant (*χ* ^2^ [120] = 3435.85, *p* < 0.001), indicating sufficient strength of correlations between the items inserted into the model. Five factors were identified from the model (Figure 2) explaining a total of 58.05% of the variance, with “F1: Familiarity with FASD” explaining 26.16%; “F2: Relevancy of FASD” explaining 10.63%; “F3: Role of PAE in FASD” explaining 7.79%; “F4: Diagnosis of FASD” explaining 6.85% of the variance, and “F5: Stigma and FASD” explaining 6.62% of the variance. Lastly, the item, “FASD occurs primarily in financially disadvantaged families” did not meaningfully load onto any factors and was thus excluded from further analyses (Table 4).

**TABLE 4:** Exploratory factor analysis on Knowledge and Attitudes Regarding FASD Measure, with oblimin rotation and maximum likelihood extraction.

Item	*α* if item deleted	F1: Familiarity with FASD	F2: Relevancy of FASD	F3: Role of PAE in FASD	F4: Recognition of FASD	F5: Stigma toward FASD
I am familiar with the difficulties people with FASD can experience	0.753	1.00	*0.037*	*0.069*	*0.0326*	*0.019*
I am familiar with how FASD can affect people's lives	0.755	0.772	*0.022*	*0.047*	*0.008*	*0.010*
I am familiar with how alcohol use during pregnancy can affect fetal development	0.765	0.651	*0.003*	*0.066*	*0.029*	*0.058*
I am not familiar with the cause of FASD^a^	0.755	0.350	*0.073*	*0.272*	*0.086*	*0.102*
FASD is only relevant to people aged under 18 years^a^	0.764	*0.064*	0.699	*0.108*	*0.112*	*0.090*
People can grow out of FASD^a^	0.758	*0.035*	0.434	*0.211*	*0.093*	*0.117*
FASD is relevant to my work	0.777	*0.109*	0.383	*0.001*	*0.078*	*0.060*
Alcohol's negative effect on fetal development has been proven	0.766	*0.052*	*0.188*	0.581	*0.081*	*0.140*
People with FASD have permanent brain damage	0.772	*0.043*	*0.059*	0.507	*0.025*	*0.094*
FASD can be diagnosed at any age	0.767	*0.206*	*0.020*	0.315	*0.004*	*0.140*
The benefits of a diagnosis of FASD do not outweigh the harm it can cause to families^a^	0.781	*0.032*	*−0.089*	*0.014*	0.590	*0.149*
Diagnosis of FASD would not improve outcomes for those affected by FASD^a^	0.765	*−0.008*	*0.215*	*0.017*	0.509	*0.183*
The emphasis on FASD is stigmatizing to women^a^	0.791	*0.035*	*0.017*	*0.008*	0.301	*0.127*
Most birth mothers who drink when pregnant know it can harm their baby^a^	0.782	*0.020*	*0.019*	*0.043*	*−0.008*	0.559
All people with FASD have particular facial characteristics^a^	0.776	*0.033*	*0.101*	*0.023*	*0.055*	0.379
*FASD occurs primarily in financially disadvantaged families* ^a^	0.778	*0.100*	*0.263*	*0.141*	*0.045*	*0.192*

*Note*: Items in italics had factor loadings <0.300.

^a^ Reverse‐scored items.

### Step 2: Confirmatory factor analyses

Next, using data from the CFA group (*n* = 898), a series of CFA models were conducted. A single‐factor solution (i.e., whereby all items load onto one latent factor) was first tested in order to compare to the model fit of the optimized factor solutions identified in the EFAs.

#### Alcohol and Pregnancy Measure

The initial single‐factor structure was found to have poor model fit, meeting none of the goodness‐of‐fit criteria (*χ* ^2^ [35] = 572.85, *p* < 0.001; CFI = 0.893, TLI = 0.862, RMSEA = 0.131 [90% confidence intervals 0.122–0.140], SRMR = 0.048, BIC = 18,057.07). In contrast, the optimized three‐factor structure was found to meet most goodness‐of‐fit criteria (*χ* ^2^ [32] = 210.11, *p* < 0.001; CFI = 0.964, TLI = 0.950, RMSEA = 0.079 [90% confidence intervals 0.069–0.089], SRMR = 0.034, BIC = 17,714.72), with ΔBIC = 342.35, suggesting a three‐factor solution is more appropriate than a single‐factor solution. On inspection of the modification indices, a large residual correlation between the item dyad, “Drinking alcohol during pregnancy can lead to life‐long disabilities in a child” and “Drinking alcohol during pregnancy can affect the unborn child” was identified (MI = 89.97). Given the significant semantic overlap, the parameter was thus freed, resulting in further improvement to model fit (*χ* ^2^ [31] = 127.90, *p* < 0.001; CFI = 0.981, TLI = 0.972, RMSEA = 0.059 [90% confidence intervals 0.049–0.070], SRMR = 0.028, BIC = 17,639.32), with ΔBIC = 85.40 relative to the three‐factor solution (see Figure S1 for CFA path diagram).

#### Knowledge and Attitudes Regarding FASD Measure

The baseline single‐factor structure was found to have poor model fit, meeting none of the goodness‐of‐fit criteria (*χ* ^2^ (90) = 1030.12, *p* < 0.001; CFI = 0.725, TLI = 0.680, RMSEA = 0.108 [90% confidence intervals 0.102–0.114], SRMR = 0.086, BIC = 34,111.66). In contrast, the optimized five‐factor structure was found to have reasonable goodness‐of‐fit criteria (*χ* ^2^ [80] = 346.65, *p* < 0.001; CFI = 0.922, TLI = 0.900, RMSEA = 0.061 [90% confidence intervals 0.054–0.068], SRMR = 0.051, BIC = 33,496.19), with ΔBIC = 615.47 providing support for the optimized five‐factor model compared to the initial one‐factor solution.

Post hoc inspection of modification indices identified two residual correlations above the >20 threshold; each involving item dyads with overlapping semantic content regarding familiarity with FASD; (i) “I am familiar with how alcohol use during pregnancy can affect fetal development” and “I am not familiar with the cause of FASD” (MI = 35.29), and (ii) “I am familiar with the difficulties people with FASD can experience” and “I am familiar with how FASD can affect people's lives” (MI = 32.19). Freeing these parameters improved model fit further (*χ* ^2^ [78] = 298.02, *p* < 0.001; CFI = 0.936, TLI = 0.914, RMSEA = 0.056 [90% confidence intervals 0.049–0.063], SRMR = 0.048, BIC = 33,461.16), with ΔBIC = 35.03 relative to the original five‐factor solution (see Figure S2 for CFA path diagram).

### Internal consistency and intercorrelations between subfactors

#### Alcohol and Pregnancy Measure

Next, merging the EFA and CFA groups data (*n* = 1797), the internal consistencies and intercorrelations between the identified subfactors of the two measures were evaluated (Table 5). Upon removing the items identified in the EFA without meaningful factor loadings, the total revised *Alcohol and Pregnancy Measure* had good internal consistency (*α* = 0.892). Moreover, subfactors within the measure had internal consistency values ranging from good (e.g., “Intoxication Acceptability”; *α* = 0.855) to approaching acceptable (e.g., “Support for PAE”; *α* = 0.644). Spearman's rho correlations between the subfactors were also found to correlate significantly with medium effect sizes (e.g., *ρ* [1797] = 0.454 [95% CIs = 0.415, 0.491], *p* < 0.001).

**TABLE 5:** Spearman's rho correlations between the Alcohol and Pregnancy Measure subfactors.

Subfactor	F1	F2	F3
F1: Intoxication acceptability (*α* = 0.855)	—	0.454***	0.352***
F2: Impact of PAE (*α* = 0.850)		—	0.463***
F3: Support for PAE (*α* = 0.644)			—

***p < 0.001.

#### Knowledge and Attitudes Regarding FASD Measure

After removing the item identified within the EFA that had no meaningful factor loadings, the revised version of the total scale was found to have acceptable internal consistency (*α* = 0.784). At the subfactor level, internal consistency ranged from good (“Familiarity with FASD”; *α* = 0.838) to poor (“Stigma toward FASD”; *α* = 0.332). Moreover, all subfactors within the *Knowledge and Attitudes Regarding FASD Measure* were found to correlate significantly with small (e.g., *ρ* [1797] = 0.132 [95% CIs = 0.085, 0.178], *p* < 0.001), to medium (e.g., *ρ* [1797] = 0.495 [95% CIs = 0.458, 0.530], *p* < 0.001) effect sizes (see Table 6 for full summary).

**TABLE 6:** Spearman's rho correlations between the Knowledge and Attitudes Regarding FASD subfactors.

Subfactor	F1	F2	F3	F4	F5
F1: Familiarity with FASD (*α* = 0.838)	—	0.400***	0.495***	0.190***	0.205***
F2: Relevancy of FASD (*α* = 0.600)		—	0.425***	0.245***	0.210***
F3: Role of PAE in FASD (*α* = 0.576)			—	0.233***	0.261***
F4: Recognition of FASD (*α* = 0.428)				—	0.132***
F5: Stigma toward FASD (*α* = 0.332)					—

***p < 0.001.

### Convergent validity

Lastly, using the measures' total and subfactor scores, convergent validity with both knowledge toward FASD and attitudes toward health advice scores was assessed (see Table 7 for full overview of correlations). In short, at the total score level, both the *Alcohol and Pregnancy Measure* and *Knowledge and Attitudes Toward FASD Measure* were found to correlate significantly with health advice attitudes with medium (*ρ* [1797] = 0.343 [95% CIs = 0.300, 0.384], *p* < 0.001) and small effect sizes (*ρ* [1797] = 0.263 [95% CIs = 0.218, 0.307], *p* < 0.001), respectively; and with knowledge of FASD with small (*ρ* [1797] = 0.107 [95% CIs = 0.059, 0.155], *p* < 0.001) to medium effect sizes (*ρ* [1797] = 0.339 [95% CIs = 0.295, 0.381], *p* < 0.001), respectively. Both the Alcohol and Pregnancy Measure and Knowledge and Attitudes Regarding FASD Measure were found to significantly correlate with a small effect size (*ρ* [1797] = 0.272 [95% CIs = 0.227, 0.315], *p* < 0.001).

**TABLE 7:** Spearman's rho correlations between the Alcohol and Pregnancy Measure, Knowledge and Attitudes Regarding FASD Measure, attitudes toward health advice, and FASD knowledge scores.

Measure	1	2	3	4	5	6	7	8	9	10	11	12
1. Total *Alcohol and Pregnancy Measure*	—	0.636***	0.846***	0.778***	0.272***	0.226***	0.233***	0.284***	0.153***	0.712***	0.343***	0.107***
2. Intoxication acceptability		—	0.454***	0.352***	0.204***	0.182***	0.169***	0.207***	0.078***	0.663***	0.278***	0.050*
3. Impact of PAE			—	0.463***	0.273***	0.220***	0.229***	0.265***	0.155***	0.660***	0.269***	0.101***
4. Support for PAE				—	0.151***	0.134***	0.152***	0.186***	0.089***	0.415***	0.259***	0.075**
5. Total *Knowledge and Attitudes Regarding FASD Measure*					—	0.769***	0.694***	0.672***	0.535***	0.258***	0.263***	0.339***
6. Familiarity with FASD						—	0.400***	0.495***	0.190***	0.205***	0.261***	0.234***
7. Relevancy of FASD							—	0.425***	0.245***	0.210***	0.188***	0.222***
8. Role of PAE in FASD								—	0.233***	0.261***	0.240***	0.237***
9. Recognition of FASD									—	0.132***	0.045	0.193***
10. Stigma toward FASD										—	0.054	0.087***
11. Health advice attitudes											—	0.105***
12. FASD knowledge												—

*p < 0.05, **p < 0.01, ***p < 0.001.

## DISCUSSION

The aim of the current study was to evaluate the psychometric properties of two measures, developed to quantify attitudes toward PAE (Peadon et al., 2011) and FASD (Passmore et al., 2018). Findings suggest that both measures show generally acceptable psychometric properties, especially after scale refinement. However, given the issues of ceiling effects, dearth of established comparison measures, and low subfactor internal consistencies, such findings should be considered preliminary rather than evidence of full psychometric validity. Nevertheless, this is the first step in establishing reliable and valid measures that can be used to capture attitudes toward PAE and FASD.

While the original 12‐item *Alcohol and Pregnancy Measure* (Peadon et al., 2011) was found to have good internal consistency when summed to a singular total score (*α* = 0.823), the exploratory factor model found an improved 10‐item version of the measure with three latent subfactors (*α* = 0.892). Further support for this factor structure was found in follow‐up confirmatory factor analyses, with the three‐factor solution showing improved model fit when compared to the baseline single‐factor solution. Regarding the scale's convergent validity, scores were found to correlate significantly with Attitudes toward Health Advice scores. In other words, if participants tended to report more informed attitudes toward alcohol use during pregnancy, they were in turn more likely to endorse the current “no alcohol, no risk” messaging (Chief Medical Officer, 2016). However, while a significant correlation was also identified between PAE attitudes and knowledge of FASD, this relationship was weak (*ρ* [1797] = 0.106). On the one hand, it could be argued that knowledge is intrinsically yoked with attitudes (i.e., poorer attitudes are a consequence of lower knowledge; Jomezadeh et al., 2023); thus, inversely, those who demonstrate greater knowledge of FASD will also show more informed attitudes toward PAE. However, given the weak correlation that emerged, knowledge of FASD and attitudes toward PAE may not be as closely linked in participants as first predicted. Indeed, past evidence has suggested while a large proportion (80%) of the general population agree that occasional alcohol use during pregnancy should be avoided, only 22% knew what the acronym FASD stood for (National Organisation for FASD, 2020). Lastly, it should be noted that a below‐acceptable internal consistency was identified within the three‐item subfactor, “Support for PAE” (*α* = 0.644). A possible cause of this is that while two items pertain to support provision for pregnant women (e.g., “Health professionals should advise women who are pregnant or who are thinking of becoming pregnant to give up drinking alcohol”), the final item evaluates the participants’ perceptions of public awareness of PAE risks (“Members of the general public are concerned about women drinking alcohol during pregnancy”). It is likely that participants, while endorsing advice provision for pregnant women, were more uncertain regarding the level of awareness that the general population holds for PAE, leading to poor internal consistency of the subfactor. Indeed, when this item is removed, the subfactor's internal consistency is above the recommended threshold of *α* > 0.700. It is therefore suggested future use of this measure exclude this item or interpret results with caution.

Similar findings were observed for the *Knowledge and Attitudes Regarding FASD Measure* (Passmore et al., 2018). After refining the original 16‐item scale via exploratory factor analysis, a five‐factor solution was found to be more appropriate when compared to a single‐factor solution, with most items loading onto conceptually meaningful subfactors. Moreover, convergent validity was observed on both health advice messaging for pregnant women, as well as FASD knowledge. However, while the total revised scale showed acceptable internal consistency (*α* = 0.784), several of the identified subfactors did not (e.g., F3: Role of PAE in FASD; *α* = 0.576). One likely explanation is some of the measure's items did not load onto semantically aligned factors (e.g., “FASD can be diagnosed at any age”), which failed to load onto the subfactor pertaining to attitudes toward the relevancy and importance of diagnoses; instead loading onto a subfactor pertaining to the attitudes toward the role of PAE in FASD. Thus, while these items shared variance with less homogenous item clusters to create statistical factors, sufficiently defined and coherent subfactors did not emerge, resulting in poor internal consistency. Taken together, it appears caution should be taken when interpreting such subfactors in future applications of this measure. While the analyses suggest that attitudes toward FASD are multidimensional in nature, the current item clusters may not offer sufficient reliability. While evidence of the measure's theoretical dimensionality was found, this appears to come with a trade‐off in measurement reliability. Thus, in the short term, it is recommended that authors utilize the whole revised version of the *Knowledge and Attitudes Regarding FASD Measure* until further refinement of the measure is achieved.

Longer term, continued work in scale development is required, especially in developing measures to capture attitudes toward FASD. A core limitation of Passmore et al.'s (2018) and Peadon et al.'s (2011) measures was the limited involvement of lived experience individuals during item development. While Peadon et al.' (2011) piloted the items in a sample of women during scale refinement, it is unclear if such women had lived experience of stigma due to prenatal alcohol use. Moreover, while Passmore et al.' (2018) included foster parents of children with FASD in their item development, they did not include any individuals with FASD themselves. This is a particular concern as such individuals would likely provide deeper insight into the potential attitudes held by others regarding their condition. Moreover, while efforts were taken in the current study to improve generalisability of the measure outside of criminal justice professionals, it is possible that certain items still were not suitable for use outside of this population; thus, poor internal consistencies were observed. It is therefore recommended that a more co‐created, generalisable measure be created and used in the future.

### Limitations

There are several limitations of note. Despite the large sample size across the analyses, data were primarily derived from professionals within the Scottish National Healthcare Service and thus the generalizability of the findings is limited. Future studies should therefore explore whether the underlying factor structures identified in the current study can be replicated in other populations (e.g., those from the general population, or in education professionals) and whether measurement invariance holds across different samples. Second, pronounced ceiling effects were observed, whereby the average scores across the psychometric measures at the total score level (Table 1) and at the individual item level (Tables S3 and S4) were largely positively skewed. Such limited between‐subject score variability may have caused poorer internal consistency and weaker factor loadings (Moret et al., 2007; Terwee et al., 2007), in turn potentially underestimating the reliability and discriminative power of the two measures. Third, self‐reported attitudinal measures may be sensitive to social desirability biases (Andersen et al., 2025). In other words, it is possible that participants, concealing their true beliefs, reported attitudes toward PAE/FASD that they believed conformed to social norms. However, given the anonymous nature of data collection across both studies, it is hoped that this limited such biases. Fourth, it is possible that while the two studies recruited from the same overarching population of NHS Scotland workers, cohort effects may have been observed. While approximately half of the analyzed data were from participants actively seeking to improve their knowledge of PAE/FASD (McDougall et al., n.d.), the other half came from a broader study evaluating the “landscape” of PAE/FASD knowledge and attitudes in NHS Scotland workers (McDougall et al., 2025). Thus, it is possible that the two samples differed on interest, motivation, or professional need to upskill in PAE/FASD. Indeed, while the webinar attendees were not given any incentive to participate outside of knowledge improvement, the NHS‐wide study instead offered participants the chance to win a gift card if they participated. Nevertheless, given that the samples were merged and randomized prior to analysis, such cohort biases may have been largely mitigated. Fifth, Haywood cases were observed in the EFA models for both of the measures, likely due to the choice in using maximum likelihood as the extraction method. While this method has been routinely recommended as the most rigorous of extraction methods (Costello & Osborne, 2005), Principal Axis Factoring (PAF) has been suggested as an alternative given its more lenient assumptions (Sürücü et al., 2022). Indeed, rerunning the exploratory factor analyses with PAF extraction resolves the observed Haywood cases (Tables S5 and S6). Thus, future replications of this study should consider alternative extraction methods to avoid such cases. Lastly, to the authors’ knowledge, no other comparable measure of attitudes toward PAE nor FASD has been developed, thus the construct validity of the two measures could not be investigated. While effort was taken by the research team to develop novel measures to assess convergent validity, a possible future direction would be to explore the convergent/discriminant validity of both the *Alcohol and Pregnancy Measure* and *Knowledge and Attitudes Regarding FASD Measure* with other measures of attitudes. For example, exploring the level of convergence between measures of attitudes toward PAE/FASD and measures of attitudes toward other potentially stigmatized health conditions and behaviors, such as smoking (e.g., via the Attitudes Toward Smoking Scale; Etter et al., 2000) or mental health problems (via the Stigma and Self‐Stigma Scales; Docksey et al., 2022), would be a fruitful line of future enquiry.

## CONCLUSION

Results highlight that refined versions of Peadon et al.'s (2011) and Passmore et al.'s (2018) measures adequately capture attitudes toward PAE and FASD, respectively. While evidence for the revised *Alcohol and Pregnancy Measure*'s psychometric validity was found in the current study, it is recommended that, in the short term, researchers utilize the revised *Knowledge and Attitudes Regarding FASD Measure* at the total‐scale level and issue caution in the scale's validity to capture true attitudes toward the condition. However, long‐term recommendations include the development of a new measure to capture attitudes held toward FASD, co‐developed with lived experience individuals, that can be applied across multiple populations (e.g., across professional and public samples).

## FUNDING INFORMATION

This study was funded by the Scottish Government Funding.

## CONFLICT OF INTEREST STATEMENT

The authors declare no conflicts of interest.

## Supporting information


Appendix S1.

## Data Availability

The data that support the findings of this study are available from the corresponding author upon reasonable request.
