# Supplementary material for: Dual psychometric evaluation of measures assessing attitudes toward prenatal alcohol exposure and fetal alcohol spectrum disorder
Source: Alcohol Clin Exp Res (Hoboken). 2026 Feb 9;50(2):e70239. doi: 10.1111/acer.70239 (PMC12886179; doi:10.1111/acer.70239)
Supplement: Supplementary file 1 — Appendix S1. [file ACER-50-0-s001.docx]

**Supplemental Materials**

**Table S1.**

*Items of Attitudes Towards Health Advice Measure (3 items).*

| **Item** | **Strongly Disagree** | **Slightly Disagree** | **Neither Agree nor Disagree** | **Slightly Agree** | **Strongly Agree** |
| --- | --- | --- | --- | --- | --- |
| 1. There is no safe level of alcohol use during pregnancy. |  |  |  |  |  |
| 2. Women should avoid alcohol when trying to become pregnant. |  |  |  |  |  |
| 3. Women should avoid alcohol when they are pregnant. |  |  |  |  |  |

**Table S2.**

*Items of Knowledge of FASD Measure (12 items).*

| **Item** | **Possible Answer(s)** |
| --- | --- |
| 1. What proportion of pregnant women report drinking alcohol at any time during their pregnancy in the UK? | 1. About 10%  2. About 20%  3. About 30%  *4. About 40%  5. I don’t know |
| 2. True or false, alcohol passes freely through the placenta? | *True  False |
| 3. True or false, alcohol can cause damage at any stage of pregnancy? | *True  False |
| 4. What is Fetal Alcohol Spectrum Disorder? | *1.The spectrum of physical, emotional, and developmental delays that can affect an individual throughout their lifetime as a result of changes to the brain structure and function following prenatal exposure to alcohol  2. The short-term physical symptoms of alcohol withdrawal that babies born to mothers who used alcohol during pregnancy experience  3. Impairments in cognitive development that occur when a baby is exposed to alcohol post-birth, either directly or through breast milk  4. An increase in alcohol consumption that accompanies post-natal depression  5. I don’t know |
| 5. True or false, FASD is found across the socio-economic status spectrum? | *True  False |
| 6. What are some of the factors that can influence the presentation of FASD? (check all that apply) | *The amount of alcohol consumed  *The gestational timing of exposure  *Maternal nutrition  *Pattern of consumption (e.g., binge drinking)  The price of alcohol consumed  I don’t know |
| 7. What is the estimated prevalence of FASD in Scotland? | Less than 1%  Between 1% and 3%  *Between 3% and 5%  More than 5%  I don’t know |
| 8. What proportion of individuals with FASD display one or more of the sentinel facial features (e.g., smooth philtrum, thin upper lip, short palpebral fissures [the horizontal opening of the eye])? | 100%  50%  *10%  1%  I don’t know |
| 9. What are some of the benefits of a diagnosis of FASD for the individuals and their families? | 1. Help ensure that individuals with FASD obtain more suitable, FASD-specific care and intervention  2. Can reduce FASD risk for future pregnancies  3. Helps families and individuals to understand the nature of the difficulties and respond appropriately  *4. All of the above  5. I don’t know |
| 10. What are some of the reasons that individuals with FASD may go undiagnosed or receive an incorrect diagnosis? (check all that apply) | *1. Lack of facial features  *2. Questions about alcohol use in pregnancy not asked  *3. Need for further training among health professionals  *4. The behavioural presentation may be similar to other neurodevelopmental impairments including ADHD and autism  5. I don’t know |
| 11. True or false, the early and accurate diagnosis of FASD reduces the risk of negative long-terms outcomes? | *True  False |
| 12. FASD may be over-represented in which of the following populations? (Check all that apply) | 1. Care experienced/looked-after children  2. Criminal justice system  3. Homelessness  4. Individuals with substance use issues  5. I don’t know |

Note. Correct answer(s) indicated with asterisk.

**Table S3.**

Means, standard deviations, and Spearman correlations, between all items within the *Alcohol and Pregnancy Measure.*

|  | **1** | **2** | **3** | **4** | **5** | **6** | **7** | **8** | **9** | **10** | **11** | **12** |
| --- | --- | --- | --- | --- | --- | --- | --- | --- | --- | --- | --- | --- |
| 1. Health Professionals should advise women who are pregnant or who are thinking of becoming pregnant to give up drinking alcohol (M = 4.33, SD = .904) | ^/^ | .523^***^ | .150^***^ | .354^***^ | .062^**^ | .399^***^ | .222^***^ | .417^***^ | .346^***^ | .381^***^ | .003 | .479^***^ |
| 2. Health professionals should ask pregnant women about how much and how often they drink alcohol (M = 4.49, SD = .824) |  | / | .210^***^ | .407^***^ | .038 | .386^***^ | .291^***^ | .415^***^ | .334^***^ | .321^***^ | .022 | .382 |
| 3. Members of the general public are concerned about women drinking alcohol during pregnancy (M = 3.69, SD = .877) |  |  | / | .202^***^ | .249^***^ | .156^***^ | .171^***^ | .147^***^ | .164^***^ | .119^***^ | .068^**^ | .091^***^ |
| 4. Information should be readily available to women about the effect that drinking alcohol may have on the unborn (M = 4.78, SD = .694) |  |  |  | / | .010 | .360^***^ | .284^***^ | .401^***^ | .353^***^ | .307^***^ | .110^***^ | .317^***^ |
| 5. Women are aware of the effects that drinking alcohol during pregnancy can have on the unborn child (M = 3.20, SD = .981) |  |  |  |  | / | .068^**^ | .132^***^ | .089^***^ | .038 | .044 | .005 | .001 |
| 6. Drinking alcohol during pregnancy can lead to life-long disabilities in a child (M = 4.50, SD = .818) |  |  |  |  |  | / | .356^***^ | .660^***^ | .348^***^ | .412^***^ | .055^*^ | .448^***^ |
| 7. The more alcohol a pregnant women drinks, the more likely that the unborn child will be affected (M = 4.42, SD = .942) |  |  |  |  |  |  | / | .471^***^ | .269^***^ | .260^***^ | .147^***^ | .250^***^ |
| 8. Drinking alcohol during pregnancy can affect the unborn child (M = 4.62, SD = .775) |  |  |  |  |  |  |  | / | .378^***^ | .425^***^ | .071^**^ | .466^***^ |
| 9. It is ok for pregnant women to become intoxicated (M = 4.69, SD = .842) |  |  |  |  |  |  |  |  | / | .610^***^ | .087^***^ | .417^***^ |
| 10. It is ok for pregnant women to drink three or four units of alcohol in one day (M = 4.57, SD = .900) |  |  |  |  |  |  |  |  |  | / | .038 | .466^***^ |
| 11. Pregnant women should drink less than seven units of alcohol each week (M = 3.66, SD = 1.61) |  |  |  |  |  |  |  |  |  |  | / | .067^**^ |
| 12. Pregnant women should not drink alcohol (M = 4.43, SD = 1.01) |  |  |  |  |  |  |  |  |  |  |  | / |

***Note.*** M = mean, SD = standard deviation.

**Table S4.**

Means, standard deviations, and Spearman correlations, between all items within the *Alcohol and Pregnancy Measure.*

|  | **1** | **2** | **3** | **4** | **5** | **6** | **7** | **8** | **9** | **10** | **11** | **12** | **13** | **14** | **15** | **16** |
| --- | --- | --- | --- | --- | --- | --- | --- | --- | --- | --- | --- | --- | --- | --- | --- | --- |
| 1. I am familiar with how alcohol use during pregnancy can affect fetal development (M = 3.71, SD = .889) | / | .625^***^ | .457^***^ | .548^***^ | .267^***^ | .242^***^ | .324^***^ | .024 | .106^***^ | .243^***^ | .160^***^ | .144^***^ | .049^*^ | .093^***^ | .199^***^ | .197^***^ |
| 2. I am familiar with the difficulties people with FASD can experience (M = 3.53, SD = .978) |  | / | .507^***^ | .762^***^ | .377^***^ | .310^***^ | .313^***^ | .117^***^ | .177^***^ | .294^***^ | .180^***^ | .209^***^ | .065^**^ | .069^**^ | .258^***^ | .218^***^ |
| 3. I am not familiar with the cause of FASD (M = .379, SD = 1.00) |  |  | / | .483^***^ | .356^***^ | .306^***^ | .386^***^ | .118^***^ | .196^***^ | .366^***^ | .178^***^ | .265^***^ | .132^***^ | .110^***^ | .236^***^ | .277^***^ |
| 4. I am familiar with how FASD can affect people's lives (M = 3.58, SD = .957) |  |  |  | / | .393^***^ | .292^***^ | .334^***^ | .095^***^ | .173^***^ | .295^***^ | .180^***^ | .213^***^ | .068^**^ | .057^*^ | .271^***^ | .261^***^ |
| 5. FASD can be diagnosed at any age (M = 3.59, SD = .753) |  |  |  |  | / | .296^***^ | .286^***^ | .122^***^ | .216^***^ | .285^***^ | .132^***^ | .217^***^ | .107^***^ | .065^**^ | .217^***^ | .240^***^ |
| 6. People with FASD have permanent brain damage (M = 3.63, SD = .770) |  |  |  |  |  | / | .380^***^ | .051^*^ | .070^***^ | .380^***^ | .065^**^ | .180^***^ | .099^***^ | .057^*^ | .139^***^ | .225^***^ |
| 7. Alcohol's negative effect on fetal development has been proven (M = 4.17, SD = .788) |  |  |  |  |  |  | / | .041 | .084^***^ | .383^***^ | .103^***^ | .211^***^ | .115^***^ | .125^***^ | .187^***^ | .365^***^ |
| 8. Most birth mothers who drink when pregnant know it can harm the baby (M = 3.03, SD = .995) |  |  |  |  |  |  |  | / | .195^***^ | .121^***^ | .137^***^ | .146^***^ | .089^***^ | .042 | .095^***^ | .049^*^ |
| 9. All people with FASD have particular facial characteristics (M = 3.25, SD = 1.07) |  |  |  |  |  |  |  |  | / | .157^***^ | .183^***^ | .219^***^ | .184^***^ | .022 | .169^***^ | .147^***^ |
| 10. People can grow out of FASD (M = 4.10, SD = .875) |  |  |  |  |  |  |  |  |  | / | .233^***^ | .312^***^ | .127^***^ | .065^**^ | .239^***^ | .464^***^ |
| 11. FASD occurs primarily in financially disadvantaged families (M = 3.46, SD = 1.02) |  |  |  |  |  |  |  |  |  |  | / |  | .086^***^ | .049^*^ | .179^***^ | .173^***^ |
| 12. Diagnosis of FASD would NOT improve outcomes for those affected by FASD (M = 3.77, SD = .923) |  |  |  |  |  |  |  |  |  |  |  | / | .349^***^ | .130^***^ | .234^***^ | .309^***^ |
| 13. The benefits of a diagnosis of FASD do not outweigh the harm it can cause to families (M = 3.27, SD = 1.00) |  |  |  |  |  |  |  |  |  |  |  |  | / | .144^***^ | .100^***^ | .123^***^ |
| 14. The emphasis on FASD is stigmatising to women (M = 3.19, SD = 1.02) |  |  |  |  |  |  |  |  |  |  |  |  |  | / | .007 | .107^***^ |
| 15. FASD is relevant to my work (M = 3.92, SD = 1.11) |  |  |  |  |  |  |  |  |  |  |  |  |  |  | / | .306^***^ |
| 16. FASD is only relevant to people age under 18 years (M = 4.32, SD = .906) |  |  |  |  |  |  |  |  |  |  |  |  |  |  |  | / |

***Note.*** M = mean, SD = standard deviation.

**Table S5.**

*Exploratory factor analysis on the Alcohol and Pregnancy Measure, with oblimin rotation and Principal Axis Factoring extraction.*

| Item | **α if Item Deleted** | **F1: Intoxication Acceptability** | **F2: Impact of PAE** | **F3: Support for PAE** |
| --- | --- | --- | --- | --- |
| It is ok for pregnant women to drink three or four units of alcohol in one day (*) | .784 | **.809** | *.188* | *.095* |
| It is ok for pregnant women to become intoxicated (*) | .787 | **.656** | *.265* | *.150* |
| Drinking alcohol during pregnancy can affect the unborn child | .774 | *.003* | **.867** | *.008* |
| Drinking alcohol can lead to life-long disabilities in a child | .776 | *.036* | **.815** | *.010* |
| The more alcohol a pregnant women drinks, the more likely that the unborn child will be affected | .787 | *.060* | **.748** | *.121* |
| Information should be readily available about the effect that drinking alcohol may have on the unborn child | .777 | *.039* | **.712** | *.224* |
| Pregnant women should not drink alcohol | .785 | *.064* | **.706** | *.088* |
| Health professionals should advise women who are pregnant or who are thinking of becoming pregnant to give up drinking alcohol | .782 | *.016* | *.197* | **.684** |
| Health professionals should ask pregnant women about how often they drink alcohol | .779 | *.070* | *.234* | **.547** |
| Members of the general public are concerned about women drinking alcohol during pregnancy | .805 | *.101* | *.068* | **.439** |
| *Pregnant women should drink less than seven units of alcohol each week* | .864 | *.061* | *.068* | *.071* |
| *Women are aware of the effects that drinking can have on the unborn child* | .826 | *.075* | *.068* | *.189* |

**Note.** (*) denotes reverse-scored items. Items in italics had factor loadings <.300.

**Table S6.**

*Exploratory factor analysis on Knowledge and Attitudes Regarding FASD Measure, with oblimin rotation and Principal Axis Factoring extraction.*

| Item | **α if Item Deleted** | **F1: Familiarity with FASD** | **F2: Relevancy of FASD** | **F3: Role of PAE in FASD** | **F4: Recognition of FASD** | **F5: Stigma towards FASD** |
| --- | --- | --- | --- | --- | --- | --- |
| I am familiar with the difficulties people with FASD can experience | .753 | **.972** | *.059* | *.005* | *.014* | *.047* |
| I am familiar with how FASD can affect people’s lives | .755 | **.770** | *.003* | *.025* | *.001* | *.054* |
| I am familiar with how alcohol use during pregnancy can affect fetal development | .765 | **.685** | *.006* | *.069* | *.024* | *.024* |
| I am not familiar with the cause of FASD (*) | .755 | **.406** | *.083* | *.089* | *.109* | *.212* |
| FASD is only relevant to people aged under 18 years (*) | .764 | *.064* | **.682** | *.084* | *.130* | *.107* |
| People can grow out of FASD (*) | .758 | *.013* | **.437** | *.104* | *.118* | *.291* |
| FASD is relevant to my work | .777 | *.092* | **.423** | *.076* | *.125* | *.018* |
| Alcohol’s negative effect on fetal development has been proven | .766 | *.052* | *.188* | **.581** | *.068* | *.179* |
| People with FASD have permanent brain damage | .772 | *.043* | *.059* | **.507** | *.025* | *.094* |
| FASD can be diagnosed at any age | .767 | *.206* | *.020* | **.315** | *.004* | *.140* |
| The benefits of a diagnosis of FASD do not outweigh the harm it can cause to families (*) | .781 | *.032* | *-.089* | *.014* | **.590** | *.149* |
| Diagnosis of FASD would not improve outcomes for those affected by FASD (*) | .765 | *-.008* | *.215* | *.017* | **.509** | *.183* |
| The emphasis on FASD is stigmatising to women (*) | .791 | *.035* | *.017* | *.008* | **.301** | *.127* |
| Most birth mothers who drink when pregnant know it can harm their baby (*) | .782 | *-.023* | *.020* | *.006* | *.050* | **.536** |
| All people with FASD have particular facial characteristics (*) | .776 | *.066* | *.103* | *.039* | *.006* | **.392** |
| *FASD occurs primarily in financially disadvantaged families (*)* | .778 | *.118* | *.261* | *.174* | *.068* | *.179* |

**Note.** (*) denotes reverse-scored items. Items in italics had factor loadings <.300.


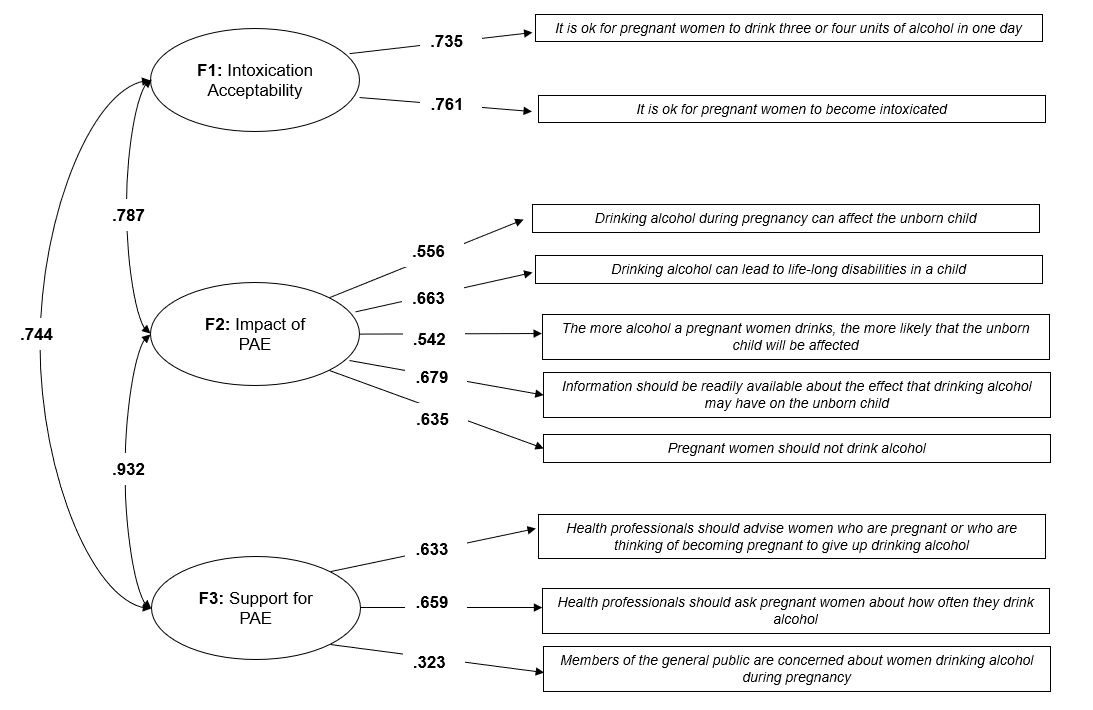


**Figure S1.** *Path diagram of confirmatory factor analysis on the Alcohol and Pregnancy Measure. Solid lines indicate significant paths. Double-headed arrows denote covariances.*


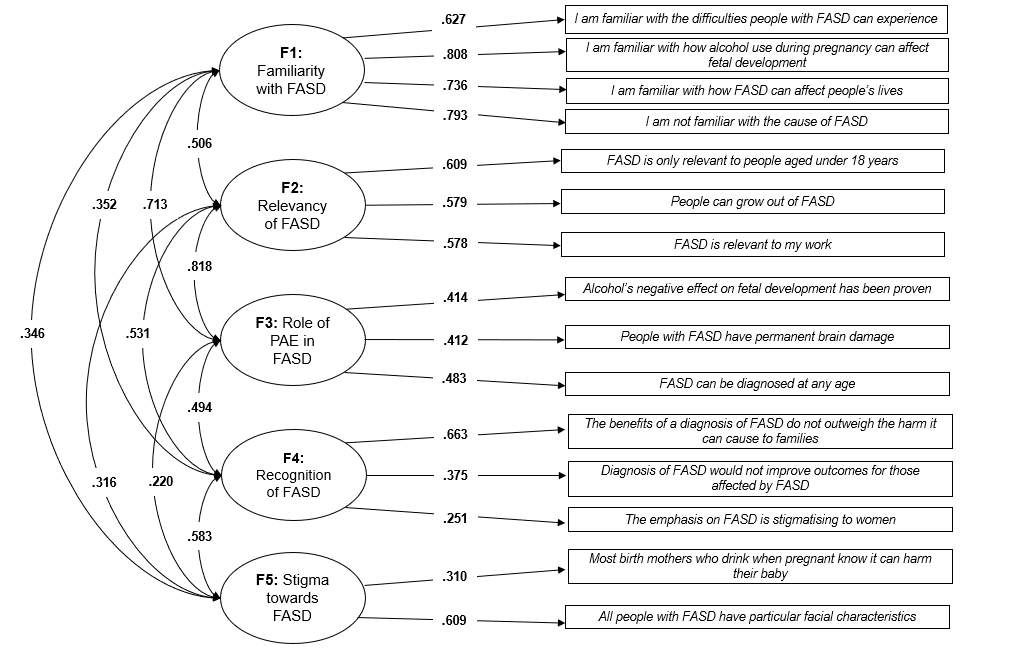


**Figure S2.** *Path diagram of confirmatory factor analysis on the Knowledge and Attitudes Regarding FASD Measure. Solid lines indicate significant paths. Double-headed arrows denote covariances.*
